# The Role of Vericiguat in Early Phases of Anterior Myocardial Infarction: A Potential Game-Changer?

**DOI:** 10.3390/medicina60101595

**Published:** 2024-09-28

**Authors:** Federico Cacciapuoti, Ciro Mauro, Valentina Capone, Salvatore Chianese, Luca Gaetano Tarquinio, Rossella Gottilla, Fabio Marsico, Salvatore Crispo, Fulvio Cacciapuoti

**Affiliations:** 1 Department of Internal Medicine, “L. Vanvitelli” University, 80131 Naples, Italy; 2 Division of Cardiology, “A. Cardarelli” Hospital, Via A. Cardarelli, 6, 80131 Naples, Italy; 3 Department of Advanced Biomedical Sciences, “Federico II” University, 80131 Naples, Italy; 4 Post-Graduate School of Emergency Medicine, “L. Vanvitelli” University, 80131 Naples, Italy

**Keywords:** anterior myocardial infarction, vericiguat, cyclic guanosine monophosphate (cGMP), nitric oxide (NO), cardioprotection

## Abstract

Anterior myocardial infarction is a critical condition with significant implications for cardiac function and patient prognosis. Despite advancements in reperfusion therapies, optimizing recovery during the early phases of myocardial infarction remains challenging. Anterior myocardial infarction can lead to substantial long-term effects on a patient’s health due to extensive damage to the heart muscle, particularly the left ventricle, impacting both quality of life and overall prognosis. Vericiguat, a soluble guanylate cyclase stimulator, has shown promise in heart failure, but its role in early anterior myocardial infarction has not yet been fully explored. By enhancing soluble guanylate cyclase activity, vericiguat may increase cyclic guanosine monophosphate production, leading to vasodilation, inhibition of platelet aggregation, and potential cardioprotective effects. Currently, treatment options for anterior myocardial infarction primarily focus on reperfusion strategies and managing complications. However, there is a critical need for adjunctive therapies that specifically target the pathophysiological changes occurring in the early phases of myocardial infarction. Vericiguat’s mechanism of action offers a novel approach to improving vascular function and myocardial health, potentially contributing to innovative treatment strategies that could transform the care and prognosis of patients with anterior myocardial infarction.

## 1. Introduction

Anterior myocardial infarction (MI) is a serious, life-threatening event characterized by the blockage of blood flow to the anterior wall of the heart, leading to significant damage to cardiac muscle. Anterior MIs are among the most severe types of heart attacks, associated with worse outcomes compared to other infarct locations, such as inferior or lateral MIs. This type of MI accounts for approximately 33% of all cases [1]. Globally, coronary artery disease (CAD), the primary cause of MI, remains the leading cause of death, with around 17.9 million deaths annually, representing approximately 31% of all global deaths [2]. A substantial fraction of these deaths is attributed to anterior MI due to its higher morbidity and mortality rates, particularly in older adults [3]. 

**Figure 1:**
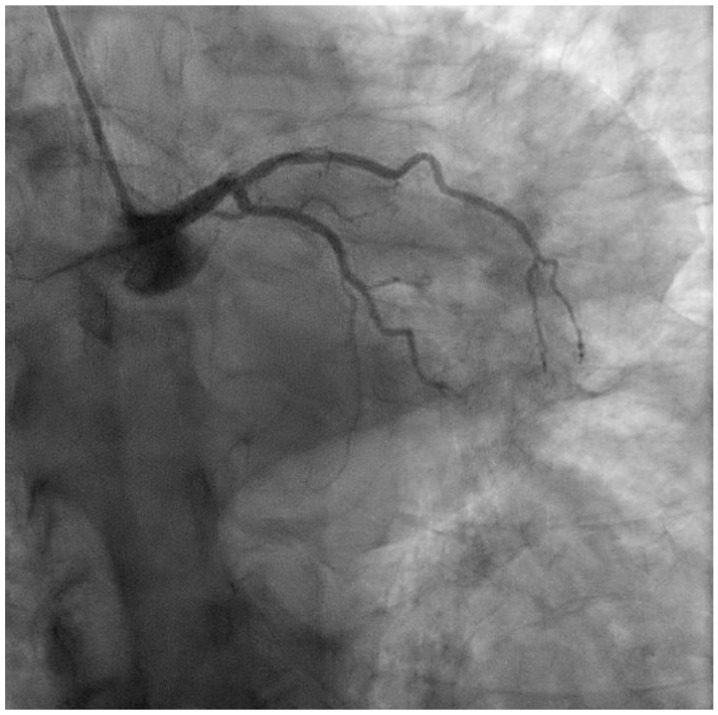
Coronary angiography performed during anterior STEMI, showing thrombotic occlusion of the ostial LAD.

In the short term, complications such as acute heart failure, arrhythmias, and cardiogenic shock can arise from substantial damage to the left ventricle, compromising its pumping ability [4]. Despite advances in acute MI management, including timely reperfusion via primary percutaneous coronary intervention (PCI) [5,6] and improved pharmacotherapy, anterior MI continues to carry a higher risk of complications and mortality compared to other MI types. Recent data indicate in-hospital mortality rates of 8–12% for anterior MI, compared to 4–6% for other types, with 30-day mortality ranging from 8 to 14% for anterior MI versus 5–8% for others [7]. This highlights the disproportionate burden of anterior MI, particularly due to the larger area of myocardial damage associated with left anterior descending (LAD) artery occlusion (Figure 1). Short- and long-term complications are significantly influenced by factors such as heart failure development (seen in up to 25% of anterior MI patients), as well as left ventricular (LV) dysfunction, remodeling, fibrosis, and an increased risk of recurrent MI (Table 1) [8]. Additionally, fibrosis and electrical instability in the damaged myocardium can lead to persistent arrhythmias, further exacerbating heart failure. While timely reperfusion therapy has effectively reduced mortality [9], additional therapeutic strategies are necessary to optimize cardiac recovery, particularly in the early phases of MI.

One potential therapeutic candidate is vericiguat, a soluble guanylate cyclase (sGC) stimulator that has shown promise in treating heart failure with reduced ejection fraction (HFrEF). The VICTORIA trial highlighted vericiguat’s role in improving outcomes for patients with HFrEF [10], making it a compelling candidate for use in acute anterior MI. However, its role in the specific setting of early anterior MI has yet to be fully explored, despite its potential to address critical pathophysiological changes and optimize recovery, thereby preventing long-term cardiac dysfunction [11].

The pathophysiology of myocardial infarction involves complex biochemical pathways, including the nitric oxide (NO)-soluble guanylate cyclase (sGC)-cyclic guanosine monophosphate (cGMP) signaling cascade [12,13]. Under healthy conditions, NO stimulates sGC, leading to increased cGMP production, a molecule that promotes vasodilation and helps regulate blood flow [14]. However, during MI, NO availability is often significantly reduced due to ischemia and endothelial dysfunction, impairing the sGC-cGMP pathway and leading to decreased cGMP production, vasoconstriction, and exacerbation of ischemic injury [15]. 

The findings from the VICTORIA trial suggest that vericiguat’s ability to enhance myocardial function by increasing cGMP production [16] may be particularly beneficial in anterior MI, where left ventricular function is often compromised. The cardioprotective effects observed in HFrEF, such as improved vascular tone, reduced myocardial remodeling, and decreased fibrosis, may also translate to better outcomes in anterior MI patients. By directly stimulating sGC independent of NO, vericiguat can bypass the impaired NO-sGC interaction [17]. This unique mechanism can lead to increased cGMP levels, even under conditions of reduced NO availability, which are typical during MI. Vericiguat’s potential to enhance vasodilation, reduce myocardial oxygen demand, and inhibit platelet aggregation presents a compelling case for its use in the early stages of MI, where swift intervention is crucial for limiting myocardial damage [18].

Further supporting this, a recent systematic review and network meta-analysis on the efficacy of modern therapies for HFrEF highlights vericiguat as a promising option across various population subgroups [19]. This study showed that vericiguat’s benefits extend beyond general heart failure management and are consistent across different patient demographics. These findings raise the possibility that vericiguat could offer similar cardioprotective benefits in the acute phase of MI, potentially reducing the progression to heart failure and other long-term complications.

## 2. Rationale

### 2.1. The Role of Nitric Oxide Deficiency in Post-Myocardial Infarction and the Therapeutic Potential of Vericiguat

Nitric oxide (NO) plays a key role in cardiovascular physiology by maintaining vascular tone, reducing inflammation, and protecting against ischemic injury [20]. However, in post-MI patients, NO bioavailability is significantly reduced due to endothelial dysfunction, oxidative stress, and the upregulation of pathways that degrade NO [21]. This deficiency contributes to increased myocardial damage, impaired vascular function, and the progression of heart failure (HF), a common and deadly complication in post-MI survivors.

NO is synthesized primarily in endothelial cells by endothelial nitric oxide synthase (eNOS). Upon release, NO diffuses into vascular smooth muscle cells, where it binds to soluble guanylate cyclase (sGC), triggering the conversion of guanosine triphosphate (GTP) to cyclic guanosine monophosphate (cGMP). Elevated cGMP levels induce vasodilation, reduce vascular resistance, and inhibit smooth muscle proliferation and platelet aggregation. This pathway is crucial for maintaining cardiac function and vascular homeostasis, especially in ischemic injury settings like MI [22]. 

In post-MI patients, the NO-sGC-cGMP pathway becomes dysregulated due to endothelial dysfunction, oxidative stress, and sGC inactivation. Reduced NO bioavailability following MI profoundly affects both the acute and chronic phases of myocardial injury. With lower NO levels, vasoconstriction becomes more prominent, worsening myocardial ischemia by reducing blood flow to already oxygen-deprived areas. Furthermore, a deficient NO system leads to endothelial dysfunction, contributing to atherosclerosis progression, poor angiogenesis, and impaired coronary blood flow regulation [23]. NO deficiency also promotes adverse ventricular remodeling, including myocardial fibrosis, ventricular dilatation, and reduced cardiac output, all worsening heart failure. A lack of NO exacerbates HF by increasing afterload, impairing myocardial contractility, and promoting the development of pulmonary hypertension [24]. Finally, NO stabilizes myocardial cell electrical activity, and its deficiency increases the risk of arrhythmias [25], a major cause of sudden death in post-MI patients.

These adverse effects highlight the critical need for therapeutic strategies that can restore or enhance NO-cGMP signaling to improve outcomes in post-MI patients. Vericiguat is an oral soluble guanylate cyclase stimulator designed to enhance cGMP production independently of NO availability. It addresses the limitations of current heart failure therapies, particularly in post-MI patients with NO deficiency, by directly stimulating sGC even in the presence of oxidative stress, which can impair NO-mediated signaling [26]. 

Vericiguat directly binds to sGC and stimulates it to produce cGMP without requiring NO. This is particularly advantageous in post-MI patients, where oxidative stress often reduces NO availability and sGC becomes less responsive to endogenous NO. Moreover, in tissues where NO is still partially available, vericiguat enhances the sensitivity of sGC to the remaining NO, amplifying its effects on cGMP production [27]. This combination of NO-independent stimulation and NO-sensitization gives vericiguat a distinct advantage over traditional vasodilators or earlier-generation sGC modulators, which rely more heavily on the presence of endogenous NO for therapeutic effects.

By stimulating cGMP production in vascular smooth muscle cells, vericiguat promotes vasodilation, reducing afterload and improving coronary blood flow. This helps mitigate ischemic injury and supports better oxygen delivery to the myocardium. Increased cGMP levels help prevent pathological myocardial remodeling by reducing fibrosis, preserving ventricular geometry, and improving myocardial contractility. Furthermore, clinical trials such as VICTORIA have demonstrated that vericiguat significantly reduces the risk of hospitalization due to heart failure [28]. This is particularly relevant for post-MI patients, who are at high risk of recurrent decompensations.

### 2.2. The Promise of Vericiguat in Early Myocardial Infarction

In the context of early anterior MI, the application of vericiguat could represent a significant therapeutic advancement. By enhancing sGC activity and increasing cGMP production, vericiguat could address critical challenges associated with MI (Table 2). Increased cGMP levels can facilitate the relaxation of vascular smooth muscle, promoting vasodilation and improving blood flow to the ischemic myocardium. This effect could reduce the extent of myocardial damage and enhance overall cardiac function. Moreover, the anti-platelet effects of cGMP could help prevent further coronary artery occlusion, thereby reducing the risk of subsequent cardiac events. 

The cardioprotective effects associated with elevated cGMP levels, such as reduced cardiac remodeling and fibrosis, are particularly relevant in the setting of MI [29]. Specifically, the guanylyl cyclase B (GC-B)/cGMP signaling pathway has consistently been reported as a potent inhibitor of organ fibrosis in various experimental models of cardiovascular diseases [30]. By mitigating fibrosis and stabilizing cardiac electrophysiology, vericiguat could aid in managing heart failure, prevent adverse cardiac remodeling, reduce the incidence of arrhythmias, and lower the risk of recurrent cardiovascular events in patients following an anterior MI. These effects could counteract adverse cardiac remodeling, a common consequence of extensive myocardial injury and a precursor to heart failure.

### 2.3. Potential Challenges and Considerations

The theoretical benefits of vericiguat in early phases of MI are promising; however, several challenges and considerations must be addressed. First, the safety profile of vericiguat in the acute MI setting requires thorough evaluation. While the drug has shown safety in chronic heart failure populations, the acute context presents unique risks. Its integration with existing therapies must be carefully considered to ensure both efficacy and safety.

One primary treatment for MI is the use of angiotensin-converting enzyme (ACE) inhibitors, which reduce the production of angiotensin II, a potent vasoconstrictor. By promoting vasodilation and decreasing afterload, ACE inhibitors play a crucial role in improving long-term survival and preventing adverse cardiac remodeling [31]. Vericiguat’s mechanism, enhancing cGMP production via the sGC pathway, also promotes vasodilation but through a different biochemical process. This complementary action could theoretically improve myocardial oxygen supply and reduce cardiac workload post-MI. However, since both ACE inhibitors and vericiguat can lower blood pressure, there is concern for additive hypotension, particularly in patients with borderline or low baseline blood pressure. This underscores the need for the careful monitoring of blood pressure and renal function when these drugs are used together.

Another critical therapy following MI is the administration of beta-blockers, which lower myocardial oxygen demand by reducing heart rate, contractility, and blood pressure. Beta-blockers also possess anti-arrhythmic properties, helping prevent life-threatening arrhythmias during the post-MI period [32,33]. While beta-blockers focus on decreasing oxygen consumption, vericiguat aims to enhance oxygen supply by promoting vasodilation and improving coronary and systemic perfusion. This combination could potentially optimize myocardial oxygen balance, offering a more comprehensive approach to post-MI care. However, similar to ACE inhibitors, beta-blockers can amplify hypotensive effects when combined with vericiguat, necessitating careful titration to avoid symptomatic hypotension. Additionally, since beta-blockers can slow heart rate, their interaction with vericiguat may pose a risk of bradycardia or excessive heart rate reduction, requiring close monitoring in clinical practice.

Vericiguat could also interact with aldosterone antagonists, often prescribed after MI to reduce heart failure progression [34]. These agents block aldosterone’s effects, reducing sodium retention and preventing adverse cardiac remodeling [35]. Vericiguat’s ability to enhance vasodilation and improve myocardial perfusion could complement aldosterone antagonists, potentially offering a dual approach to minimizing heart failure progression. However, both vericiguat and aldosterone antagonists may contribute to hyperkalemia, making potassium level monitoring crucial for patients receiving both therapies.

In addition to heart failure-focused treatments, antiplatelet therapy, often in the form of dual antiplatelet therapy (DAPT) with aspirin and P2Y12 inhibitors, is essential following MI to prevent thrombosis [36,37]. Vericiguat’s ability to increase cGMP levels may also confer anti-platelet effects by inhibiting platelet aggregation, providing an additional protective mechanism against clot formation. While this synergy might enhance thrombo-protection, there is a risk of increased bleeding when combining vericiguat with potent antiplatelet agents. Clinicians must carefully assess the bleeding risk in individual patients before combining these therapies.

Vericiguat may also play a beneficial role in patients undergoing reperfusion therapy, such as percutaneous coronary intervention (PCI) or thrombolytic therapy. By promoting vasodilation and improving microvascular flow, vericiguat could enhance the effects of reperfusion, helping to preserve myocardial tissue after large coronary arteries are reopened. Additionally, its ability to reduce platelet aggregation and vasoconstriction may help prevent no-reflow phenomena, where microvascular blockages persist despite successful reperfusion. While no significant direct interactions between vericiguat and reperfusion therapies are anticipated, there is potential for cumulative vasodilatory effects, which could lead to hypotension in some patients. 

Moreover, diuretics are often used after MI, especially in patients with heart failure, to relieve fluid overload and reduce pulmonary congestion [38]. Vericiguat’s vasodilatory effects could complement diuretics by reducing afterload and venous congestion, further alleviating symptoms of heart failure. However, combining these therapies could increase the risk of volume depletion and hypotension, necessitating close monitoring of electrolytes and kidney function.

Ultimately, while vericiguat offers potential benefits in enhancing vasodilation, improving myocardial oxygen supply, and reducing adverse remodeling after MI, its use in combination with standard MI treatments requires careful consideration. The risks of hypotension, electrolyte imbalances, and drug interactions must be closely managed.

To effectively assess the impact of vericiguat in this patient population, it is crucial to carefully select high-risk individuals, such as those experiencing cardiogenic shock [39]. Including these patients will provide valuable insights into the potential benefits or harms of this therapy in critically ill populations, particularly those with higher TIMI, GRACE, and Intermountain Risk Scores (IMRSs) [40].

Future studies are needed to explore these potential interactions in greater depth to determine how vericiguat can be safely integrated into current MI treatment protocols, optimizing outcomes while minimizing risks for patients.

## 3. Conclusions

**Figure 2:**
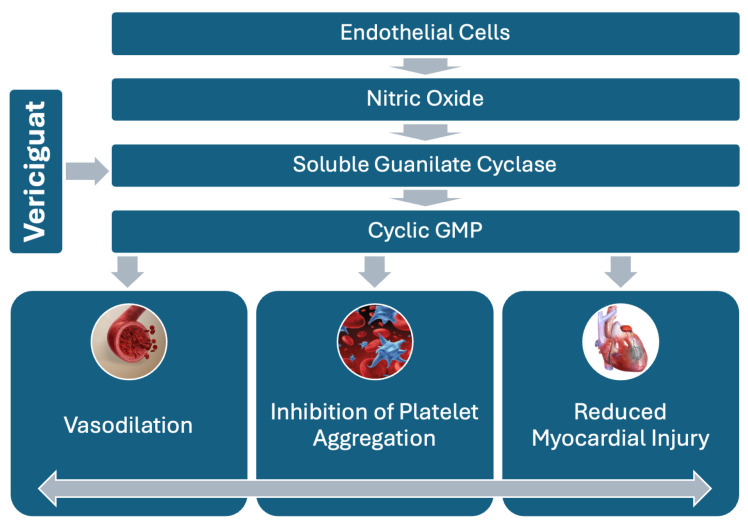
NO-sGC-cGMP pathway, highlighting vericiguat’s role in stimulating sGC to increase cGMP production. This leads to vasodilation and reduced platelet aggregation, offering therapeutic benefits in myocardial infarction.

The application of vericiguat in the early phases of anterior myocardial infarction (MI) could represent a novel approach with the potential to transform the management of this critical condition. By targeting the nitric oxide (NO)-soluble guanylate cyclase (sGC)-cyclic guanosine monophosphate (cGMP) pathway, vericiguat may improve outcomes by promoting vasodilation, reducing myocardial injury, and preventing adverse cardiac remodeling (Figure 2). While the promise of vericiguat is evident, further research is necessary to fully understand its benefits, optimize its use, and ensure safety in the acute MI setting.

Should vericiguat demonstrate efficacy and safety in this context, it could pave the way for larger-scale trials and ultimately establish a new standard of care in the management of anterior MI. In the ongoing battle against myocardial infarction, vericiguat could emerge as a valuable addition to the therapeutic arsenal, offering hope for improved outcomes in a high-risk patient population.

## Figures and Tables

**Table 1:** Early and late complications of anterior myocardial infarction (MI) along with the potential beneficial effects of vericiguat.

Complication	Timing	Description	Potential Beneficial Effects of Vericiguat
Cardiogenic Shock	Early	A severe drop in blood pressure and cardiac output due to extensive myocardial damage.	Vericiguat may improve cardiac output by enhancing myocardial contractility and vasodilation, thereby reducing afterload.
Arrhythmias (e.g., Ventricular Tachycardia, Ventricular Fibrillation)	Early	Abnormal heart rhythms due to electrical instability of the damaged myocardium.	Increased cGMP production may stabilize myocardial cell membrane potentials, reducing the risk of arrhythmias.
Acute Heart Failure	Early	Sudden onset of heart failure symptoms due to reduced left ventricular function.	By improving vasodilation and reducing cardiac preload and afterload, vericiguat may alleviate heart failure symptoms.
Pericarditis	Early	Inflammation of the pericardium, often presenting with chest pain.	Potential reduction in inflammatory response through improved endothelial function.
Left Ventricular Thrombus	Early	Formation of a blood clot in the left ventricle due to stasis of blood.	Vericiguat’s inhibition of platelet aggregation could reduce thrombus formation.
Left Ventricular Aneurysm	Late	Abnormal bulging of the ventricular wall due to scar tissue formation and remodeling.	Vericiguat may limit adverse remodeling and reduce the risk of aneurysm formation by promoting healthier myocardial repair.
Heart Failure (Chronic)	Late	Progressive decline in heart function, leading to symptoms of fluid retention and reduced exercise tolerance.	Long-term improvement in cardiac function and reduced remodeling through enhanced cGMP levels may slow the progression of heart failure.
Ventricular Septal Rupture	Early	A tear in the septum between the left and right ventricles, leading to a shunt.	Indirect benefits through overall reduction in myocardial stress and improved healing.
Mitral Regurgitation	Early	Leakage of blood backward through the mitral valve due to papillary muscle dysfunction or rupture.	Improved myocardial function may reduce the risk of mitral valve complications.
Dressler’s Syndrome	Late	A form of post-MI pericarditis occurring weeks to months after MI.	Anti-inflammatory effects of improved endothelial function may mitigate Dressler’s syndrome.
Recurrent Myocardial Infarction	Late	New myocardial infarction occurring after the initial event.	Vericiguat’s anti-platelet effects could reduce the risk of subsequent coronary events.

**Table 2:** Potential beneficial effects of vericiguat in the early phases of anterior myocardial infarction.

Beneficial Effect	Description
Enhanced Vasodilation	Vericiguat stimulates sGC, leading to increased cGMP production, which relaxes vascular smooth muscle and improves blood flow to the ischemic myocardium.
Inhibition of Platelet Aggregation	Elevated cGMP levels reduce platelet aggregation and adhesion, helping to prevent thrombus formation and further coronary artery occlusion.
Reduction in Myocardial Oxygen Demand	By promoting vasodilation and improving myocardial perfusion, vericiguat may help balance oxygen supply and demand in the heart.
Cardioprotection	Vericiguat’s effects on cGMP levels can reduce cardiac remodeling and fibrosis, which are critical factors in the prevention of heart failure following MI.
Improvement in Left Ventricular Function	Increased cGMP production can lead to improved left ventricular ejection fraction and global longitudinal strain, indicating better heart function.
Anti-inflammatory Effects	By modulating cGMP levels, vericiguat may reduce inflammatory responses, which are often exacerbated in the setting of MI.
Reduction in Vascular Resistance	Increased cGMP levels can decrease vascular resistance, potentially lowering the workload on the heart and improving overall cardiac function.

## Data Availability

No new data were created or analyzed in this study. Data sharing is not applicable to this article.
